# Colorectal Cancer in Elderly Patients: Insights into Presentations, Prognosis, and Patient Outcomes

**DOI:** 10.3390/medicina60121951

**Published:** 2024-11-26

**Authors:** Walid Shalata, Alexander Gluzman, Sofia Man, Ahron Yehonatan Cohen, Ashraf Abu Jama, Itamar Gothelf, Lena Tourkey, Ala Eddin Neime, Ali Abu Juma’a, Keren Peri-Hanania, Oshri Machluf, Gal Shoham Levin, Sondos Shalata, Ahab Hayadri, Ez El Din Abu Zeid, Nashat Abu Yasin, Amichay Meirovitz, Alexander Yakobson

**Affiliations:** 1 The Legacy Heritage Cancer Center, Dr. Larry Norton Institute, Soroka Medical Center, Beer-Sheva 84105, Israel; 2 Medical School for International Health, Faculty of Health Sciences, Ben-Gurion University of the Negev, Beer Sheva 84105, Israelabuzeid@post.bgu.ac.il (E.E.D.A.Z.); 3 Goldman Medical School, Faculty of Health Sciences, Ben-Gurion University of the Negev, Beer-Sheva 84105, Israel; gothelf@post.bgu.ac.il; 4 PhaseV Trials Ltd., Tel Aviv 67443, Israel; kren@phasevtrials.com (K.P.-H.);; 5 Nutrition Unit, Galilee Medical Center, Nahariya 22000, Israel

**Keywords:** colorectal cancer, elderly, prognostic factors, survival, diagnostic symptoms, retrospective study

## Abstract

*Background and Objectives*: Colorectal cancer (CRC) ranks as the third most prevalent cancer globally and is the third leading cause of cancer-related deaths. In 2020 alone, there were over 1.9 million new cases of CRC and nearly 0.9 million deaths worldwide. The incidence and outcomes of CRC exhibit significant geographical and temporal variations, largely influenced by diverse risk factors among populations. Recognizing the prognostic factors and the presenting symptoms of CRC, a leading global cancer with high mortality, can enhance early detection and thereby improve clinical outcomes. *Materials and Methods*: This retrospective, observational study analyzed 724 CRC elderly patients aged 70 and over (median age 80, 53.17% male), treated at a single center. Data on demographics, clinical characteristics, and outcomes were collected. Overall survival was analyzed using Kaplan–Meier curves, with stratification based on tumor location, disease staging, lymph node involvement, and family history. *Results*: Our study encompassed all CRC cases treated with surgery and systemic therapies (chemotherapy or biological agents) from July 2002 to September 2020. We focused on comparing prognosis between left-sided and right-sided CRC, as well as rectal cancer. We found that left-sided CRC demonstrated a superior prognosis compared to rectal cancer (*p* = 0.0022). Furthermore, among patients with CRC, tumors originating in the rectum were associated with worse outcomes compared to those arising in both the right and left colon, regardless of disease stage (*p* = 0.0049). Additionally, a family history of CRC was associated with poorer prognosis, impacting both metastatic (*p* = 0.0022) and localized disease (*p* = 0.035). The main symptoms prompting patients to start an investigation of CRC were abdominal pain (31.49%), anemia (18.08%), rectal bleeding (hematochezia) (17.82%), change in bowel habits (9.94%), and weight loss (7.60%). *Conclusions*: This study provides valuable insights into the symptoms prompting initial investigation and the prognostic factors associated with CRC in an elderly population with varied characteristics. It underscores the need for increased vigilance in recognizing key symptoms and the importance of personalized treatment strategies tailored to these prognostic factors.

## 1. Introduction

Colorectal cancer (CRC) is not only one of the most frequently diagnosed cancers globally but also ranks as the third leading cause of cancer-related deaths worldwide, regardless of gender. According to the World Health Organization (WHO), over 1.9 million new cases of CRC and more than 930,000 deaths were reported in 2020. This disease is influenced by a combination of environmental factors and genetic predispositions and the incidence of colorectal cancer increases with age, in which people older than 50 years face a particularly high risk, constituting more than 90% of all CRC cases. Additionally, CRC incidence is higher in men compared to women [1,2,3,4,5,6].

In recent decades, several risk factors have been identified as contributors to the incidence of CRC, with lifestyle factors playing a prominent role. Obesity, which has surged to epidemic proportions, is recognized as a significant risk factor, increasing CRC risk by 1.2 to 1.5 times in overweight individuals and by 1.5 to 1.8 times in those with obesity. Alcohol consumption, smoking, and a high intake of red and processed meat are also linked to increased CRC incidence, with alcohol raising the risk nearly 1.79 times and smoking and red meat consumption contributing to a 0.16 to 0.22 increase. Conversely, several observational studies suggest that physical activity is associated with a reduced risk of CRC. The risk reduction ranges from 0.30 to 0.50 [5,6,7,8,9,10].

Additionally, symptoms such as hematochezia (blood in the stool) and abdominal pain, along with changes in bowel habits affecting over 25% of patients, are associated with an increased risk of CRC, particularly hematochezia, which is linked to at least a five-fold increase in CRC risk [9,10,11,12].

CRC typically begins in the epithelial cells of the colorectal mucosa, progressing through stages of hyperplasia, atypical hyperplasia, and adenomas before developing into carcinoma. The survival outcomes can vary significantly based on the location and size of the primary tumor in the colon; notably, right-sided colon cancers tend to exhibit lower net survival rates compared to left-sided colon cancers. Around 80% of newly diagnosed colorectal cancer (CRC) patients are initially diagnosed with non-metastatic disease, meaning the cancer cells are localized within the primary tumor and have not spread to other organs. The preferred treatment for these patients typically involves radical surgery to completely remove the malignant tumor, which is considered the standard approach [1,2,3,12,13,14,15,16].

Advances in screening tools, chemotherapy drugs, radiotherapy equipment, and surgical techniques have together led to a decreasing annual risk of cancer recurrence following surgery. These advancements have notably enhanced outcomes for non-metastatic CRC patients, significantly lowering the chances of cancer returning after surgical intervention [17,18,19,20].

However, despite these advances, approximately 30 to 50% of colorectal cancer (CRC) patients still experience disease recurrence following radical surgery. Particularly concerning is that about 25% of patients diagnosed with theoretically curable stage I and II disease eventually suffer a recurrence. The recurrence of cancer significantly impacts patient prognosis: for those who remain free of recurrence one year after surgery, the 5-year death rate related to CRC is only 3.8%, contrasting sharply with 33.6% for those experiencing recurrence [12,13,14,15,16,17,18].

The decision to administer adjuvant chemotherapy hinges on the risk of recurrence, primarily determined by the pathological staging of the tumor. Stage I CRC patients typically face a recurrence rate of 3–5%, whereas stage II patients face a higher risk ranging from 10 to 17%. Stage III CRC patients bear the highest risk, with recurrence rates ranging from 31 to 40%, often experiencing disease relapse earlier than patients in earlier stages. The median durations until recurrence after surgery are approximately 22.6 months for stage I, 18.2 months for stage II, and 15.9 months for stage III (lymph node (LN) involvement) CRC patients. As a result, several CRC guidelines strongly advocate for adjuvant chemotherapy in stage III patients while deeming it unnecessary for stage I patients. Stage II patients are selectively considered for adjuvant chemotherapy, mainly those identified as high-risk for recurrence [15,16,17,18,19,20].

Given the high recurrence rates despite advancements in CRC treatment and the critical role of pathological staging in determining patient outcomes, this study aims to further explore the impact of various prognostic factors—tumor location, disease staging, lymph node involvement, and family history—on improving survival rates among CRC patients.

## 2. Materials and Methods

### 2.1. The Design of the Study

This study is a retrospective, real-world observational analysis conducted at a single center, focusing on patients diagnosed with local disease, locally advanced, or metastatic CRC. This study was conducted according to the guidelines of the Declaration of Helsinki and approved by the Institutional Review Board of Soroka Medical Center on colon cancer among elderly people: prevalence, patient characteristics, and risk factors. This study was approved by the Institutional Review Board of Soroka Medical Center (approval no. 0202, on 2 June 2021). Patient recruitment was based on electronic and documented medical records, encompassing all CRC cases treated with surgery and systemic therapies (chemotherapy or biological agents) from July 2002 to September 2020.

The data collected included patient demographics such as age and sex, outcomes like mortality (Yes/No) and date of death, symptoms that lead to diagnosis, lymph node involvement, smoking history (if applicable, packs per year), presence of inflammatory bowel disease, family history of cancer, obesity (defined as a Body Mass Index (BMI) greater than 30), tumor location (right-sided, left-sided, or rectal), and blood test results at diagnosis (including white blood cell count, neutrophils, lymphocytes, monocytes, platelets, hemoglobin, and hemoglobin A1C). Other morbidities were identified based on a standardized list of diagnoses using the International Classification of Diseases, Ninth and Tenth Revision (ICD-9 or ICD-10) codes. All information was retrieved from medical records and databases to analyze survival outcomes (Table 1).

### 2.2. Study Population and Inclusion Criteria

This study included patients aged 70 years and older who were diagnosed with local, locally advanced, or metastatic colorectal cancer. The designation of individuals aged 70 and above as “elderly” is widely accepted in the medical literature, reflecting increased life expectancy and evolving societal perspectives on aging. Studies and clinical guidelines frequently employ 70 as a threshold for defining “elderly” status, particularly in CRC research, as over 60% of CRC patients are over 70—a proportion expected to increase in the coming years [21,22]. Patients were either treated exclusively at Soroka Medical Center or had a comprehensive follow-up history documented in the center’s records. Upon admission to Soroka Medical Center’s Oncology Institute, each patient underwent a thorough evaluation and assessment by a multidisciplinary medical team consisting of oncologists, surgeons, radiologists, nuclear physicians, and pathologists. The treatment plan was planned based on the patient’s medical condition, pathology findings, and imaging results, under the supervision of a primary physician responsible for managing the treatment course. Patients previously diagnosed with advanced or metastatic disease primarily received care from medical oncologists, adhering to the guidelines set forth by the National Comprehensive Cancer Network (NCCN) [23], with routine molecular profiling conducted whenever feasible.

### 2.3. Exclusion Criteria

The exclusion criteria encompassed patients with incomplete data that could significantly alter the information as well as those under 70 years of age.

### 2.4. Data and Statistical Analysis

Descriptive statistics were calculated for all parameters in this study, including percentages, ranges, means, medians, and interquartile ranges (IQRs). The study parameters comprised both numerical data (e.g., OS in months and age in years) and binary data (e.g., smoking, obesity, and inflammatory bowel disease, Yes/No or 1/0). Descriptive statistics summarized the treatment outcome measures, including adverse events (AEs), employing the median (m), OS, range, frequencies (n), OS, and percentages.

Kaplan–Meier analysis was utilized to assess overall survival rates in the study population. The analysis included the stratification of the study population into subgroups based on different characteristics such as tumor location (right-sided, left-sided, or rectal), disease stage (localized or metastatic), lymph node involvement, family history of cancer, and others. The Restricted Mean Survival Time (RMST) was calculated and compared across these different subgroups. The log-rank test was used to test the hypothesis of a survival difference between the subgroups within each stratified analysis. Finally, the Benjamini–Hochberg procedure was performed to derive multiplicity-adjusted *p*-values, with a significance threshold set at a *p*-value of 0.05. The statistical analysis was conducted by Phase V Trials Ltd. (Tel Aviv, Israel).

## 3. Results

In this study, 724 patients diagnosed with colorectal cancer were included, with a median age of 80 years (range 70–97) and a mean follow-up duration of 89.6 months. When broken down by gender, male patients had a median age of 80 years (range 70–97) while female patients had a median age of 80 years (range 71–91). Regarding gender distribution among the patients, there were 385 males (53.17%) and 339 females (46.83%). The majority of patients were non-smokers at the time of diagnosis, totaling 404 individuals (55.80%), while 320 patients (44.20%) reported being smokers.

A minority of patients had a history of inflammatory bowel disease (IBD) at the time of diagnosis, comprising 33 cases (4.56%). The vast majority of patients, 691 individuals (95.44%), did not have a history of IBD. Family history of colorectal cancer was reported in 195 patients (26.93%) while 520 patients (71.82%) had no family history and 9 patients (1.24%) had an unknown family history.

In terms of obesity at diagnosis, 90 patients (12.43%) were classified as obese, while the majority, 634 patients (87.57%), were not obese. Among those diagnosed with colorectal cancer, 173 patients (36.97%) had local disease with lymph node involvement, whereas 295 patients (63.03%) did not.

Examining the staging at diagnosis, 497 patients (80.53%) had localized disease, while 120 patients (19.47%) presented with metastatic cancer. The distribution of the primary cancer location revealed that 285 patients (40.09%) had cancer in the left colon, 263 patients (37.03%) in the right colon, and 163 patients (22.88%) in the rectum.

The main symptoms that led to investigations and subsequent diagnosis varied among patients. Abdominal pain was the most common symptom reported, noted in 228 patients (31.49%). Other significant symptoms included anemia in 131 patients (18.08%), rectal bleeding in 129 patients (17.82%), and changes in bowel habits in 72 patients (9.94%). Weight loss was reported by 55 patients (7.60%), constipation by 54 patients (7.46%), and fecal occult blood test positivity by 30 patients (4.14%). Fewer patients reported diarrhea (nine patients, 1.24%), follow-up for polyps (nine patients, 1.24%), and a family history of colorectal cancer (seven patients, 0.97%) as the main reasons for initiating investigations.

In a marginal analysis of overall survival in the full cohort using a Cox Proportional Hazards (PH) model, age at diagnosis was associated with reduced overall survival (HR = 1.13, 95% CI: 1.10–1.15, *p* < 0.005).

**Figure 1:**
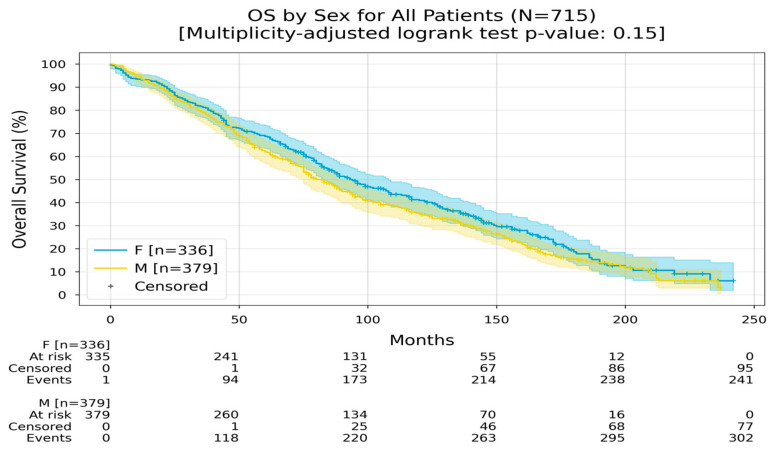
Kaplan–Meier curves with 95% CI for overall survival of all colorectal cancer patients. The multiplicity-adjusted *p*-value of the log-rank statistic for survival difference between the groups was 0.0022.

Figure 1 depicts overall survival by sex for all colorectal cancer patients over a follow-up period of up to 250 months. Kaplan–Meier curves indicate slightly higher survival in female patients compared to male patients though the difference is not statistically significant (multiplicity-adjusted log-rank test *p* = 0.15).

**Figure 2:**
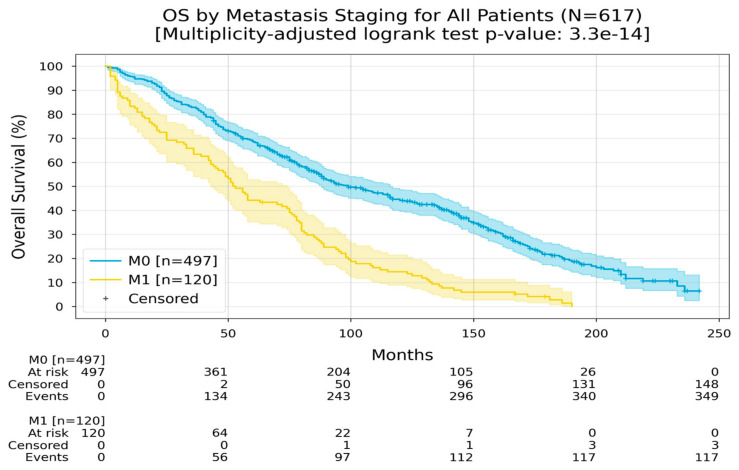
OS by metastasis staging Kaplan–Meier curves with 95% CI for overall survival, stratified by metastasis staging (M0, M1). In the M0 group, the Restricted Mean Survival Time (RMST) at 240 months was 113.4 months; in the M1 group, the RMST at 240 months was 63 months. The multiplicity-adjusted (BH) *p*-value of the log-rank statistic for survival difference between the groups was <0.001.

In the comparison between patients with metastatic and non-metastatic disease, those without metastases had a Restricted Mean Survival Time (RMST) of 113 months, whereas patients with metastatic disease had an RMST of 63 months (Figure 2).

**Figure 3:**
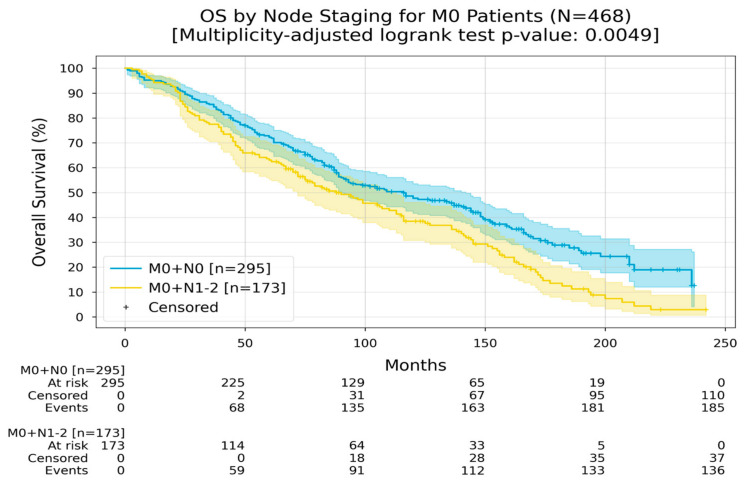
Kaplan–Meier curves with 95% CI for overall survival of M0 patients, stratified by node staging (M0 + N0, M0 + N1-2). In the M0 + N0 group, the Restricted Mean Survival Time (RMST) at 240 months was between 124 and 124.3 months; in the M0 + N1-2 group, the RMST at 240 months was 100.1 months. The multiplicity-adjusted (BH) *p*-value of the log-rank statistic for survival difference between the groups was 0.0049.

Regarding lymph node involvement at diagnosis for non-metastatic disease, patients without lymph involvement had an RMST of 124 months, while patients who had an involvement of lymph nodes had an RMST of 100 months (Figure 3).

**Figure 4:**
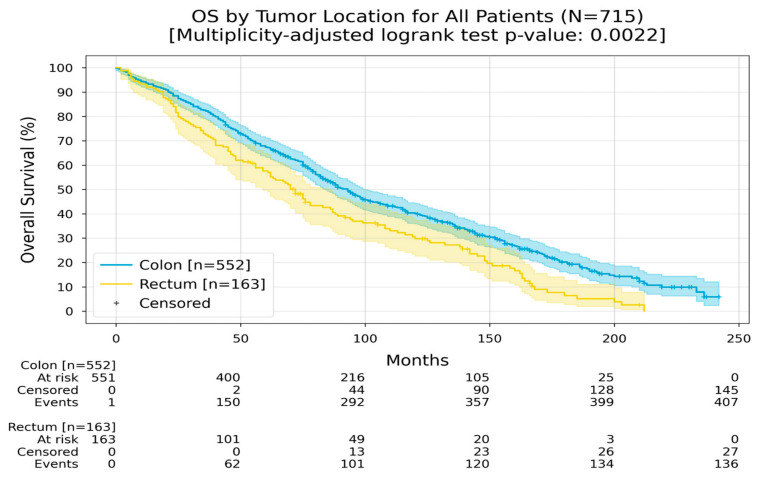
Kaplan–Meier curves with 95% CI for overall survival, stratified by tumor location (colon, rectum). In the colon group, the Restricted Mean Survival Time (RMST) at 240 months was 108.1 months; in the rectum group, the RMST at 240 months was 85.1 months. The multiplicity-adjusted (BH) *p*-value of the log-rank statistic for survival difference between the groups was 0.0022.

Location can dictate prognosis, and it was found that patients who had their primary in the colon had an RMST of 108 months, while the patients who had primary rectal cancer had an RMST of 85 months (Figure 4).

**Figure 5:**
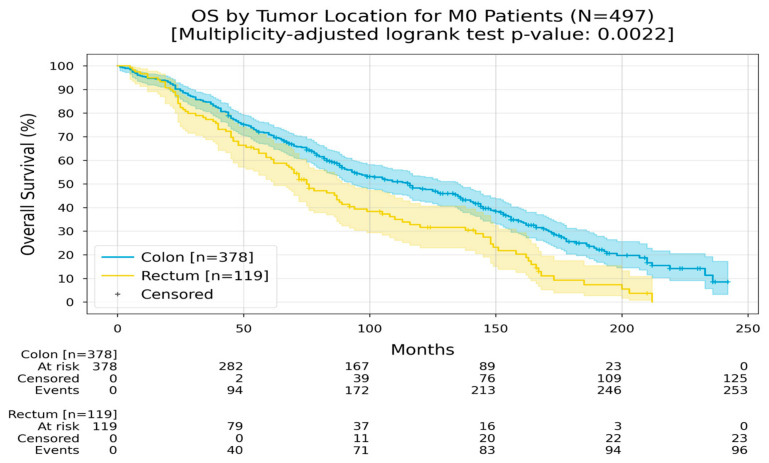
Kaplan–Meier curves with 95% CI for overall survival of M0 patients, stratified by tumor location (colon, rectum). In the colon group, the Restricted Mean Survival Time (RMST) at 240 months was 120.3 months; in the rectum group, the RMST at 240 months was 91.4 months. The multiplicity-adjusted (BH) *p*-value of the log-rank statistic for survival difference between the groups was 0.0022.

Furthermore, in patients with non-metastatic disease in the colon, the RMST was 120 months, while in the non-metastatic disease in the rectum, the RMST was 91 months (Figure 5).

**Figure 6:**
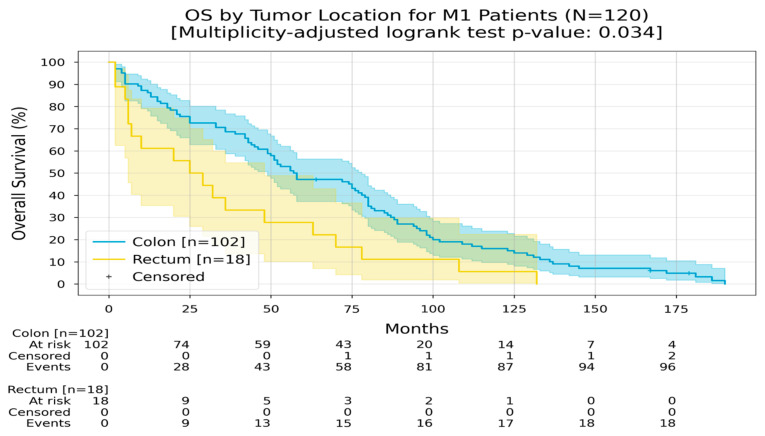
Kaplan–Meier curves with 95% CI for overall survival of M1 patients, stratified by tumor location (colon, rectum). In the colon group, the Restricted Mean Survival Time (RMST) at 240 months was 67.5 months; in the rectum group, the RMST at 240 months was 37.7 months. The multiplicity-adjusted (BH) *p*-value of the log-rank statistic for survival difference between the groups was 0.034.

In the case of a metastatic disease originating from the colon, the RMST was 67 months, while in the metastatic disease originating from the rectum, the RMST was 37 months (Figure 6).

**Figure 7:**
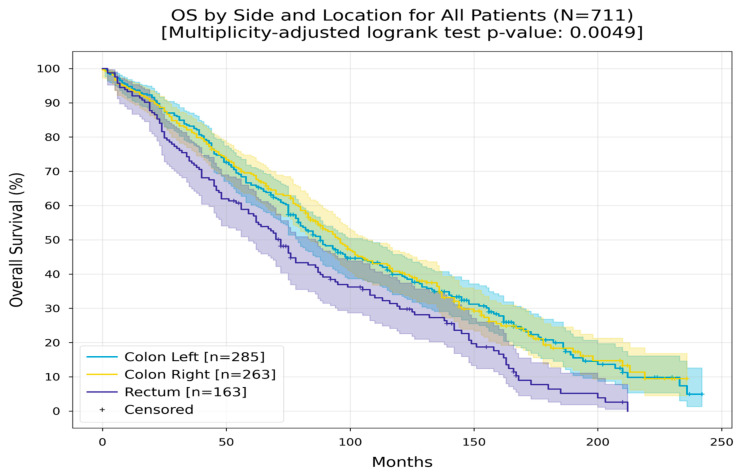
Kaplan–Meier curves with 95% CI for overall survival, stratified by side and tumor location (colon left, colon right, rectum). In the colon left group, the Restricted Mean Survival Time (RMST) at 240 months was 107.4 months; in the colon right group, the Restricted Mean Survival Time (RMST) at 240 months was between 108.5 and 108.8 months; in the rectum group, the RMST at 180 months was 85.1 months. The multiplicity-adjusted (BH) *p*-value of the log-rank statistic for survival difference between the groups was 0.0049.

Patients were stratified by tumor location into three groups: left colon, right colon, and rectum. The RMST was 107 months for patients with left-sided colorectal cancer, 108 months for those with right-sided tumors, and notably lower at 85 months for the rectal cancer group (Figure 7).

**Figure 8:**
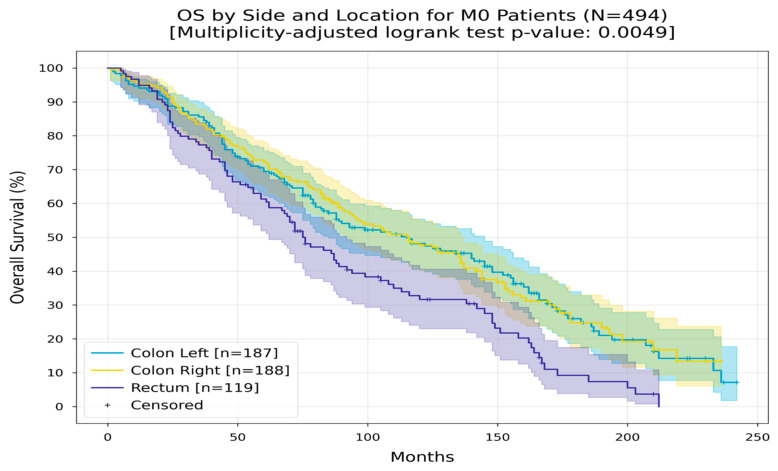
Kaplan–Meier curves with 95% CI for overall survival of M0 patients, stratified by side and tumor location (colon left, colon right, rectum). In the colon left group, the Restricted Mean Survival Time (RMST) at 240 months was 119.5 months; in the colon right group, the Restricted Mean Survival Time (RMST) at 240 months was between 120.3 and 120.8 months; in the rectum group, the RMST at 180 months was 91.4 months. The multiplicity-adjusted (BH) *p*-value of the log-rank statistic for survival difference between the groups was 0.0049.

Among patients without metastases, those with left-sided colorectal cancer had an RMST of 119 months, while those with right-sided colorectal cancer had an RMST of 120 months. Patients with non-metastatic rectal cancer had a reduced RMST of 91 months (Figure 8).

**Figure 9:**
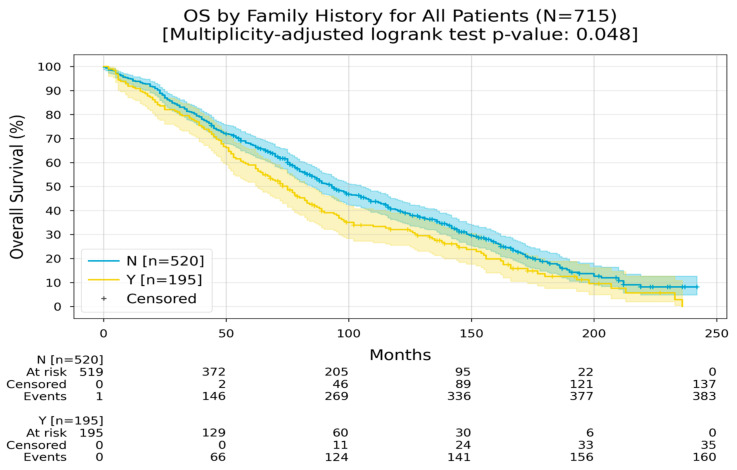
Kaplan–Meier curves with 95% CI for overall survival, stratified by family history (No, Yes). In the “No” group, the Restricted Mean Survival Time (RMST) at 240 months was 107 months; in the “Yes” group, the RMST at 240 months was 91.9 months. The multiplicity-adjusted (BH) *p*-value of the log-rank statistic for survival difference between the groups was 0.048.

Patients with a family history of colorectal cancer (CRC) had an RMST of 92 months, whereas those without a family history of CRC had an RMST of 107 months (Figure 9).

**Figure 10:**
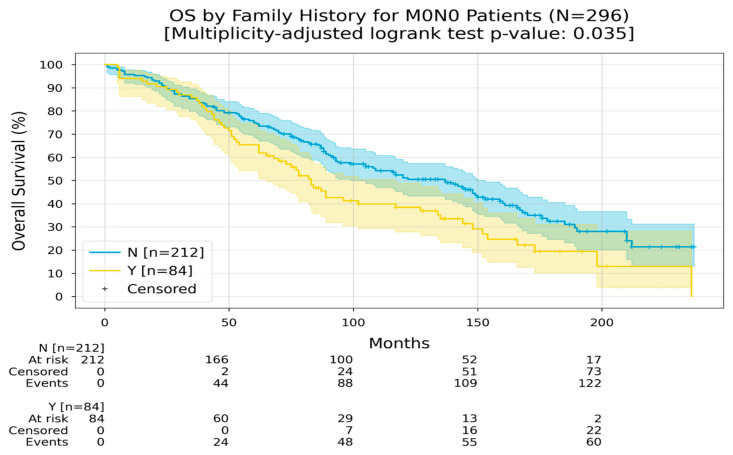
Kaplan–Meier curves with 95% CI for overall survival of M0N0 patients, stratified by family history (No, Yes). In the “No” group, the Restricted Mean Survival Time (RMST) at 240 months was between 131.1 and 131.7 months; in the “Yes” group, the RMST at 240 months was 105.5 months. The multiplicity-adjusted (BH) *p*-value of the log-rank statistic for survival difference between the groups was 0.035.

Patients with non-metastatic CRC without lymph node involvement and a negative family history had an RMST of 131 months in comparison to 105 months for those who had a positive family history (Figure 10).

**Figure 11:**
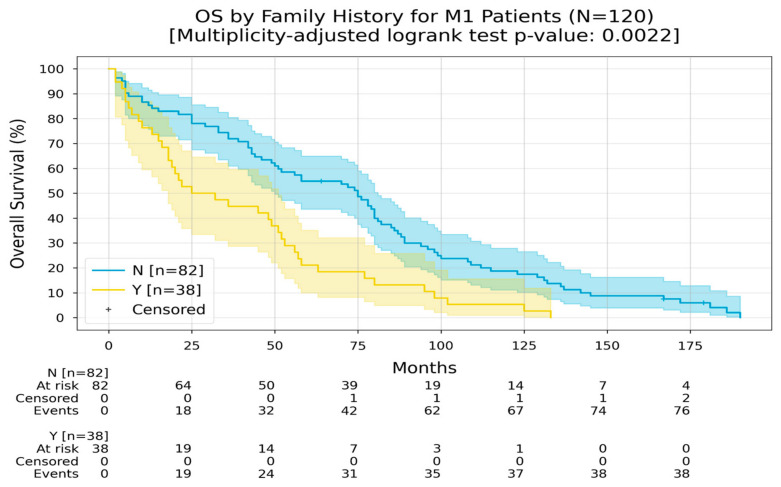
Kaplan–Meier curves with 95% CI for overall survival of M1 patients, stratified by family history (No, Yes). In the “No” group, the Restricted Mean Survival Time (RMST) at 240 months was 73.4 months; in the “Yes” group, the RMST at 240 months was 40.7 months. The multiplicity-adjusted (BH) *p*-value of the log-rank statistic for survival difference between the groups was 0.0022.

In metastatic cases, patients with a negative family history had an RMST of 73 months compared to 40 months for those with a positive family history (Figure 11).

## 4. Discussion

In Israeli society, CRC ranks third in cancer prevalence, trailing breast cancer in women and prostate cancer in men. Annually, approximately 3200 new cases of colorectal cancer are diagnosed. Over the past 27 years, early detection rates have nearly doubled, increasing from 17% to 30%. This current rate is 51% higher than the 2000 early diagnosis rate of 19.9% [5,6,24,25,26,27,28].

According to the latest data from the National Cancer Registry in 2017, Israel recorded 2312 cases of colorectal cancer, with 1153 cases in men and 1159 in women. Of these cases, 2178 were colon cancer and the remainder were rectal cancer. In 2017, colorectal cancer was the third most common invasive cancer among Jewish men (12%), following prostate and lung cancers, and the second most prevalent among Jewish women (10.2%), following breast cancer. Among Arab men, it ranked second (13.1%) after lung cancer, and among Arab women, second (9.1%) after breast cancer [5,6,24,25,26,27,28].

Age-adjusted incidence rates per 100,000 in 2017 were 27.0 for Jewish men, 22.5 for Jewish women, 28.4 for Arab men, and 18.5 for Arab women. Rates were higher in other population groups, with 44.4 for men and 27.3 for women. Incidence rates rise notably with age, peaking in the 75+ age group [5,6,26,27,28,29,30].

Based on the available data and rankings, Israel ranks relatively low in colorectal cancer (CRC) incidence rates compared to other OECD (Organisation for Economic Co-operation and Development) countries, positioned 35th out of 36 countries. A comparison and analysis estimate of male annual percentage changes with age-standardized rate (ASR) per 100,000 between Israel and other countries showed the following: Higher ASR per 100,000 compared to Israel: Countries like Denmark, Croatia, The Netherlands, and Norway have higher ASRs for CRC among males compared to Israel’s ASR of 20.1. This suggests a potentially higher incidence of CRC in these countries compared to Israel [5,6,24,25,26,27,28,29].

Similar ASR to Israel: Israel’s ASR of 20.1 is comparable to countries like Germany (20.4), Italy (21.1), and the United Kingdom (ranging from 20.8 to 23.1) [5,6,29].

Lower ASR compared to Israel: countries such as Chile (5.8), Ecuador (11.0), Colombia (10.3), and Thailand (9.9) have notably lower ASRs for CRC among males compared to Israel [5,6,29].

And for females: Higher ASR compared to Israel: Countries like Iceland, Denmark, Norway, and New Zealand have higher ASRs for CRC among females compared to Israel’s ASR of 17.2. This suggests a potentially higher incidence of CRC in these countries compared to Israel [5,6,29].

Similar ASR to Israel: Israel’s ASR of 17.2 is similar to countries like Malta, the UK (England), and Ireland, indicating comparable levels of CRC incidence among females [5,6,29].

Lower ASR compared to Israel: countries such as Turkey, Thailand, Ecuador, and Bahrain have notably lower ASRs for CRC among females compared to Israel [5,6,29].

These comparisons highlight the variability in CRC incidence rates across different countries, influenced by factors such as healthcare infrastructure, screening practices, geographical factors, lifestyle factors, and genetic predisposition

When comparing our findings with results from a recent meta-analysis systematic review involving a large cohort of 24,908,126 patients, notable differences and similarities in symptom presentation and risk factors leading to colorectal cancer (CRC) diagnosis emerge [9], and similar results have been found in other studies [30,31,32,33].

In a previous meta-analysis and systematic review, it was shown that hematochezia (blood in stool) was prevalent in 45% of cases, while abdominal pain and altered bowel habits were reported in 40% and 27% of cases, respectively. Anemia was observed in 2.1% of cases. These symptoms underscored significant indicators leading to the diagnosis of CRC in younger individuals. Additionally, factors such as a family history of CRC, which increases the incidence by two to four times, and personal histories of adenomatous polyps and inflammatory bowel disease (IBD) were identified as crucial risk factors for CRC [9,30,31,32,33].

In contrast, our study revealed a different distribution of symptoms among CRC patients. Abdominal pain was the most frequently reported symptom, accounting for 31.49% of cases. Anemia and hematochezia followed closely, noted in 18.08% and 17.82% of patients, respectively. Changes in bowel habits, weight loss, and constipation were also prominent symptoms reported in our dataset. Less frequently observed symptoms included positive fecal occult blood tests, diarrhea, and polyps follow-up, each affecting a smaller percentage of patients. Family history of colorectal cancer was relatively rare, noted in only 0.97% of cases in our study population.

Comparing these findings highlights both similarities and differences in symptom prevalence and the significance of risk factors across different studies and patient populations. While hematochezia and abdominal pain are consistently recognized as significant symptoms across studies, their prevalence rates can vary. Our study emphasizes the high frequency of abdominal pain and anemia compared to the systematic review, which highlighted altered bowel habits more prominently. Both datasets underscore the critical role of family history, adenomatous polyps, and IBD as important risk factors for identifying individuals at higher risk for CRC.

Limitations of this study include the following: Single institution: this study was conducted at a single institution, which may limit the generalizability of the findings to broader populations or different healthcare settings. Age restriction: Only patients above 70 years old were included in this study. This age restriction could introduce selection bias and limit the applicability of this study’s conclusions to younger age groups who may have different disease characteristics or outcomes. Retrospective study design: This study relied on retrospective data collection, which may be susceptible to inherent biases such as incomplete medical records, retrospective reporting errors, and the inability to establish causal relationships. Retrospective studies also lack the ability to control variables and may not capture all relevant data compared to prospective studies.

## 5. Conclusions

This study provides valuable insights into the symptoms prompting initial investigation and the prognostic factors associated with CRC in an elderly population with varied characteristics. The findings underscore the pivotal influence of tumor location, metastasis stage, and family history on patient outcomes. These results emphasize the necessity of heightened vigilance in recognizing critical symptoms in elderly individuals at risk and the importance of developing personalized treatment approaches that incorporate these essential prognostic factors in the management of CRC. By adopting this approach, clinicians can more accurately stratify risk and implement tailored treatment strategies that effectively address the distinct needs of this vulnerable population.

## Figures and Tables

**Table 1:** Baseline disease characteristics of the study population (*n* = 724).

		Median (Range)
**Age (years)**		80 (70–97)
	Male	80 (70–97)
	Female	80 (71–91)
**Gender**		**Frequencies (Percentage)**
	Male	385 (53.17)
	Female	339 (46.83)
**Smoking status (at diagnosis)**		
	Yes	320 (44.20)
	No	404 (55.80)
**Inflammatory bowel disease**		
	Yes	33 (4.56)
	No	691 (95.44)
**Family history**		
	Yes	195 (26.93)
	No	520 (71.82)
	Unknown	9 (1.24)
**Obesity (at diagnosis)**		
	Yes	90 (12.43)
	No	634 (87.57)
**Local disease with lymph node involvement (at diagnosis)**		
	Yes	173 (36.97)
	No	295 (63.03)
**Staging (at diagnosis)**		
	Local	497 (80.53)
	Metastatic	120 (19.47)
**Side of primary cancer**		
	Left colon	285 (40.09)
	Right colon	263 (37.03)
	Rectum	163 (22.88)
**Main symptom that led to start investigations**		
	Abdominal pain	228 (31.49)
	Anemia	131 (18.08)
	Rectal bleeding (hematochezia)	129 (17.82)
	Change in bowel habits	72 (9.94)
	Weight loss	55 (7.60)
	Constipation	54 (7.46)
	Positive fecal occult blood test	30 (4.14)
	Diarrhea	9 (1.24)
	Polyps follow up	9 (1.24)
	Family history of colorectal cancer	7 (0.97)

## Data Availability

The data either reside within the article itself or can be obtained from the authors upon making a reasonable request.
